# *Wolbachia*-Based Population Control Strategy Targeting *Culex quinquefasciatus* Mosquitoes Proves Efficient under Semi-Field Conditions

**DOI:** 10.1371/journal.pone.0119288

**Published:** 2015-03-13

**Authors:** Célestine M. Atyame, Julien Cattel, Cyrille Lebon, Olivier Flores, Jean-Sébastien Dehecq, Mylène Weill, Louis Clément Gouagna, Pablo Tortosa

**Affiliations:** 1 CRVOI, Ste Clotilde, Réunion Island, France; 2 University La Réunion, Réunion Island, France; 3 MIVEGEC—UMR 5290-224, CNRS-IRD-UM1-UM2, Montpellier, France; 4 UMR PVBMT, CIRAD, St Pierre, Réunion Island, France; 5 ARS-OI, St Denis, Réunion Island, France; 6 CNRS, ISEM—UMR 5554-UM2, Montpellier, France; Centro de Pesquisas René Rachou, BRAZIL

## Abstract

In mosquitoes, the maternally inherited bacterial *Wolbachia* induce a form of embryonic lethality called cytoplasmic incompatibility (CI). This property can be used to reduce the density of mosquito field populations through inundative releases of incompatible males in order to sterilize females (Incompatible Insect Technique, or IIT, strategy). We have previously constructed the LR[*w*Pip(Is)] line representing a good candidate for controlling field populations of the *Culex quinquefasciatus* mosquito in the islands of the south-western Indian Ocean. The main purpose of the present study was to fill the gap between laboratory experiments and field implementation, *i*.*e*. assessing mating competitiveness of these incompatible males under semi-field conditions. In a first set of experiments, we analyzed crossing relationships between LR[*w*Pip(Is)] males and La Réunion field females collected as larvae in 19 distinct localities throughout the island. This investigation revealed total embryonic mortality, confirming the strong sterilizing capacity of LR[*w*Pip(Is)] males. Subsequently, mating competitiveness of LR[*w*Pip(Is)] males was assessed under semi-field conditions in the presence of field males and females from La Réunion. Confrontations were carried out in April and December using different ratios of LR[*w*Pip(Is)] to field males. The results indicated that the LR[*w*Pip(Is)] males successfully compete with field males in mating with field females, displaying even higher competitiveness than field males in April. Our results support the implementation of small-scale field tests in order to assess the feasibility of IIT against *Cx*. *quinquefasciatus* in the islands of southwestern Indian Ocean where this mosquito species is a proven competent vector for human pathogens.

## Introduction

Mosquito-borne diseases such as malaria, dengue, chikungunya and Rift valley fever are among the leading causes of mortality and morbidity in humans. In the absence of effective vaccines, the control of mosquito natural populations is one of the few available strategies for limiting pathogen transmission to humans. Considerable efforts have been made in order to control mosquito natural populations notably through the use of insecticides providing outstanding results among which the eradication of malaria in several countries after World war II [1]. However, the recurrent selection of resistant individuals in response to frequent and often suboptimal implementation of insecticides together with the potential negative effects of insecticides on non-targeted organisms are a problem of clearly growing concern [2, 3]. More recently, the development of a number of insecticide free strategies has emerged with the aim of providing alternative or at least complementary tools for the control of vector populations. Among these strategies, the use of the endosymbiotic bacteria *Wolbachia* has been focusing increasing attention and is currently under development in several countries worldwide [4–7].

*Wolbachia* are maternally inherited bacteria widespread in filarial nematodes and arthropods [8]. These bacteria also infect some mosquito species of medical importance such as the members of the *Culex pipiens* complex [9, 10] and the Asian tiger mosquito *Aedes albopictus* [11]. In mosquitoes, *Wolbachia* induce a form of embryonic mortality called cytoplasmic incompatibility (CI) [12]. This phenomenon results from sperm-egg incompatibility that occurs when *Wolbachia*-infected males mate with uninfected females or with females infected with an incompatible *Wolbachia* strain. Cytoplasmic incompatibility provides this endosymbiont with strong invasive properties in field populations [13, 14]. Cytoplasmic incompatibility can be either bidirectional when the death of embryos is observed in both reciprocal crosses, or unidirectional when one cross is incompatible while the reciprocal cross is viable. In addition to the CI phenotype, previous studies have shown that *Wolbachia* infections may inhibit the development of pathogens in mosquitoes [15–19]. Inhibition of pathogens replication and strong invasive capability make *Wolbachia* a promising tool for the control of pathogens transmission through mosquito population replacement [20]. Alternatively, *Wolbachia*-induced CI can be exploited to reduce mosquito population densities *via* a derivative of the sterile insect technique (SIT) called the incompatible insect technique (IIT). This last strategy relies on the inundative releases of incompatible males, which are able to copulate and thereby sterilize females in the wild [21–23]. Incompatible insect technique was first deployed in 1967 in Burma as a measure against the filariasis vector *Cx*. *quinquefasciatus*, demonstrating the ability of *Wolbachia*-infected insects to eliminate local mosquito populations [24]. More recently, encouraging results have also been obtained in field assays against the Polynesian tiger mosquito *Aedes polynesiensis* in the South pacific islands [6] as well as in laboratory experiments targeting the medfly *Ceratitis capitata* [23], the mosquitoes *Cx*. *quinquefasciatus* [4] and *Ae*. *albopictus* [5].

*Culex pipiens* complex mosquitoes- whose main subspecies are *Culex quinquefasciatus* and *Culex pipiens*, ubiquitous in tropical and temperate regions, respectively [25, 26], are naturally infected by *Wolbachia* strains (*w*Pip) that belong to a unique clade of the B supergroup [9, 27–29]. However, *w*Pip strains display a high genetic polymorphism at a small evolutionary scale and five *w*Pip groups (referred to as *w*Pip-I to V) are currently recognized [29, 30]. Interestingly, very low *Wolbachia* diversity was found in natural populations of *Cx*. *quinquefasciatus* in the five investigated islands of south-western Indian Ocean (SWIO), namely La Réunion Island, Madagascar, Mayotte, Mauritius and Grande Glorieuse: all identified *Wolbachia* strains belonged to the *w*Pip-I group [4, 31]; thus suggesting that a single incompatible *Wolbachia* may be used for the control of all regional populations. Laboratory crossing experiments identified the *Wolbachia w*Pip(Is) strain (from the *w*Pip-IV group) as a good candidate for IIT in the islands of the SWIO. This *Wolbachia* strain was further introgressed into the nuclear background of *Cx*. *quinquefasciatus* mosquitoes from La Réunion island leading to the LR[*w*Pip(Is)] line conferring total embryonic lethality in crosses between LR[*w*Pip(Is)] males and field females sampled at a single site on each of the five investigated SWIO islands [4]. Confrontations carried out in small cages under laboratory conditions also revealed a good mating competitiveness of LR[*w*Pip(Is)] males with La Réunion field males and demonstrated that a population crash could be reached in competition experiments involving 1:5 to 1:10 La Réunion:LR[*w*Pip(Is)] males ratios [4].

In this study, we performed tests under semi-field conditions in order to assess the feasibility of IIT against *Cx*. *quinquefasciatus* in the islands of SWIO, where this species is considered as the main vector for lymphatic filarial and Rift Valley Fever virus [32], and exhibits high levels of insecticides resistance [33, 34]. As the efficacy of IIT may be impaired by the co-circulation of several distinct CI phenotypes in natural populations [31, 35], we first examined CI properties of the LR[*w*Pip(Is)] line in reciprocal crosses involving field mosquitoes sampled as larvae in 19 distinct localities on La Réunion Island. We then assessed incompatible males’ competitiveness under semi field conditions, an investigation that is required prior to open field release since these conditions are more closely related to mosquitoes’ natural habitat. Indeed, laboratory conditions are homogeneous in terms of temperature, relative humidity and light intensity; while field conditions are highly dynamic and diverse, which could affect LR[*w*Pip(Is)] males competitiveness. Overall, this study provides data that can be used for operational implementation of IIT against *Cx*. *quinquefasciatus* in SWIO islands.

## Methods

### Mosquito collections

**Fig 1:**
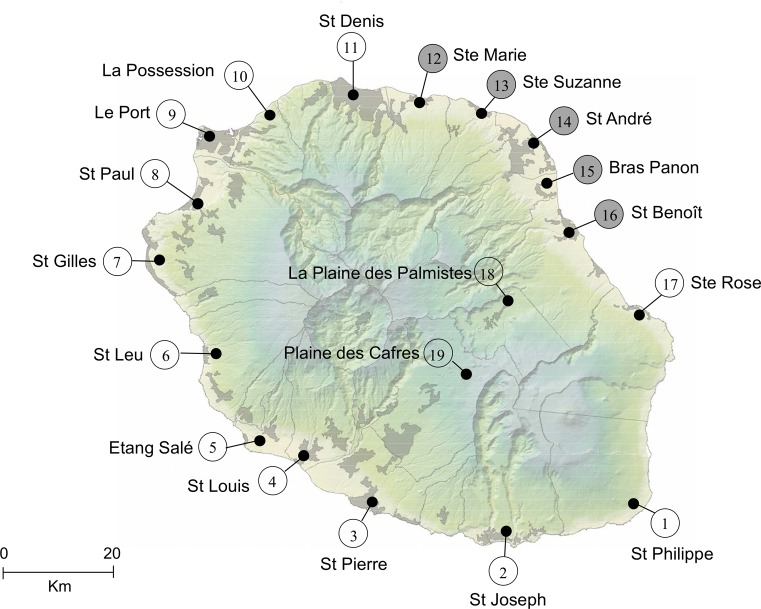
Sample sites of *Culex quinquefasciatus* field populations in La Réunion Island. Localities in grey correspond to those sites where mosquitoes were sampled for semi-field tests.

*Culex quinquefasciatus* larvae and pupae were collected in breeding sites in 19 localities of La Réunion Island in March, April, May and June 2012; and in March, April, November and December 2013 (Fig. 1). Mosquitoes were reared in the laboratory until emergence and adults were used in crossing experiments. Several hundred field-caught four instar larvae (L4) and pupae were sampled from five arbitrarily chosen localities (Ste Marie, #12; Ste Suzanne, #13; St André, #14; Bras Panon, #15 and St Benoît, #16; Fig. 1) and mosquitoes from the five populations were mixed in order to provide the field mosquitoes required for the semi-field tests. The previously described incompatible LR[*w*Pip(Is)] line, harboring the sterilizing *w*Pip(Is) strain together with a nuclear background of La Réunion mosquitoes [4] was used for the production of incompatible males.

Mosquitoes were maintained in the laboratory under conditions at 25 ± 2°C and 75 ± 2% relative humidity and a LD 12:12 h photoperiod. Larvae were reared at a density of approximately 500 larvae per tray (30×40 cm) containing 1 liter of water and were fed *ad libitum* with a mixture of rabbit and fish-food whilst adults were fed with 10% sucrose solution [w/v].

### Ethics statement

None of the samples used in this study were collected in protected areas and the *Cx*. *quinquefasciatus* mosquito is not considered as an endangered or protected species. So, no specific permission was required to collect mosquito larvae and pupae in public areas.

### Crossing experiments

Reciprocal crosses were performed in the laboratory between the LR[*w*Pip(Is)] line and field mosquitoes from 19 localities of La Réunion island (Fig. 1). Crosses were carried out in cages (30×30×30 cm) with 100–200 virgin females and an equivalent number of virgin males. All individuals were 2–5 days old (age was assessed from the emergence of adults; day 0 = emergence). Females were allowed to blood feed 5 days after caging and egg rafts (with 50–250 eggs per raft) were collected and stored individually at 25°C ± 2°C until hatching. Hatching rates (HR) were scored 72 h after egg rafts’ collection and all unhatched egg rafts were checked for embryonic development following a procedure described by Duron & Weill [36]. All unfertilized (*i*.*e*. unembryonated) egg rafts were removed from the analyses.

### Semi-field experiments

#### Field cages design

Semi-field tests were conducted on La Réunion Island within the CIRAD research institute located in La Bretagne, Saint Denis (20°90′55''S 55°49′78''E). Two set of experiments were performed: the first one in April 2013, and the second in December 2013, corresponding to the end and beginning of the warm humid season, respectively. Four field cages (180×150×150 cm) were installed and covered with gardening tents (300×300×245 cm) in order to prevent accidental escapes/invasions of mosquitoes and to protect the cages from rain (S1 Fig.). The site was partly covered with trees belonging to four species: *Inga laurina*, *Pongamia pinnata*, *Senna siamea* and *Tabebuia pallida*, providing partial protection from direct sunlight and wind. Within each field cage, a wooden table was used as resting area for mosquitoes. The bottom of table legs was placed into cups filled with water in order to prevent ants from reaching sucrose solution contained in plastic cups, which were placed on top of the table. Temperature and relative humidity were monitored using a Hobo data logger (U23–001, Pro v2) placed inside the cages. In the course of the experiments, temperatures were between 23.35°C and 26.85°C in April (mean 24.93 ± 0.16°C) and between 24.13°C and 28.18°C in December (mean 26.06 ± 0.14°C); and the values of relative humidity were between 63.48% and 80.62% in April (mean 73.70 ± 0.59%) and between 64.61% and 89.90% in December (mean 73.73 ± 0.96%) (S2 Fig.).

#### Mating competitiveness experiments

To be as close as possible to an operational phase of IIT, experiments were performed using field males and females that emerged from field collected larvae and/or pupae from five localities (Ste Marie, #12; Ste Suzanne, #13; St André, #14; Bras Panon, #15 and St Benoît, #16), whilst LR[*w*Pip(Is)] males were obtained from the mosquito line reared in standardized laboratory conditions since 2010 (*i*.*e*. during ~60 generations assuming 12 generations per year). Males and females were held in separate laboratory cages (30×30×30 cm) before being transported to the field cages where males were released before females. Only one-day-old virgin adult mosquitoes were used in the experiments. Two hundred field females were mixed with each of the four following ratios of field to LR[*w*Pip(Is)] males: 1:0 (N = 200 males), 1:1 (N = 400 males), 1:5 (N = 1200 males) and 0:1 (N = 200 males). A total of 15 trials were performed (S1 Table). These included: (a) two trials for the 1:0 and 0:1 ratios (in April), (b) six trials for the 1:1 ratio (two in April and four in December), and (c) five trials for the 1:5 ratio (two in April and three in December). For each trial, cages were randomly assigned to different locations to avoid a potential bias due to environmental variations in cage locations.

Mosquitoes released in field cages were recaptured five days following releases. Surviving mosquitoes were collected using a mouth aspirator at the end of each confrontation. Males and females were then placed into separate laboratory cages (30×30×30 cm) and brought back to the laboratory. Males were immediately stored in 70% EtOH and surviving mosquitoes were next counted and genotyped (see below); while females were blood fed in the laboratory and sugar fed until oviposition. Egg rafts were then collected and analysed as described above. Mating competitiveness of LR[*w*Pip(Is)] males was assessed by comparing the observed and expected frequencies of infertile egg rafts assuming random mating.

#### Measure of males’ survival

Survivorship can be measured by analyzing surviving males from the field cage at the end of the confrontation (e.g. see [37]) or by counting daily survival for a single category of males in individual field cages placed in the semi field setup (e.g. see [38]). Here, we used both strategies to compare the survival of field and LR[*w*Pip(Is)] males. For males recaptured from cage confrontations, we measured the survival rate by counting surviving males when only one type of males was present in cages (*i*.*e*. in the 1:0 and 0:1 control ratios) and by genotyping *Wolbachia* infecting each male’s category to determine the ratio of field *vs*. LR[*w*Pip(Is)] surviving males at the end of confrontations. Due to the high number of males released in the 1:5 ratio (N = 1200), this analysis was restricted to individuals from trials with the 1:1 ratio. The discrimination of *Wolbachia* strains infecting field males (carrying *w*Pip-I group strains) and LR[*w*Pip(Is)] males (infected with a *w*Pip-IV group strain) was performed through the genotyping of the *Wolbachia ank2* marker, an ankyrin domain encoding gene which allows distinguishing *w*Pip-I and *w*Pip-IV groups on the basis of the size of the PCR amplified fragments (313 bp and 511 bp fragments for groups I and IV, respectively [29, 39]). Estimation of daily survival of males was performed for the experiments conducted in December 2013. Freshly-emerged field (N = 220) and LR[*w*Pip(Is)] (N = 177) virgin males were introduced into separated laboratory cages (45×45×45 cm) and were transported in the semi-field setup. Males were fed with 10% sucrose solution and dead mosquitoes were checked twice a day. Survival data were fitted to the Cox proportional hazards models (*coxph*, survival package [40]) and a ratio for each type of males was estimated as their instantaneous risk of death relative to each other. These analyses were performed using R software [41].

### Data analysis

In semi-field experiments, the deviation between observed proportions of infertile egg rafts and expected proportions was tested against the null hypothesis of no deviation, as expected under random mating and equal males’ mating competitiveness hypotheses, with a Wilcoxon rank sum test. A generalized linear model (GLM) with binomial error was calibrated to test the effect of the male ratio and the period of testing on the proportion of infertile egg rafts. Male survival was compared across types (field *vs*. LR[*w*Pip(Is)]) with Kruskal-Wallis tests. All statistical analyses were performed using the R software [41].

## Results

### Stability of CI and sterilizing capacity induced by LR[wPip(Is)] males in La Réunion island

As several studies have shown that CI is a very dynamic process in the *Cx*. *pipiens* complex [31, 35, 42], we performed extensive crossing experiments between the LR[*w*Pip(Is)] line and field *Cx*. *quinquefasciatus* specimens sampled in 19 localities in La Réunion Island (Fig. 1) in order to confirm CI properties of the LR[*w*Pip(Is)] line over a wide range of natural populations. Full compatibility (HR >90%) was found in control crosses between La Réunion field males and females as well as in the cross between LR[*w*Pip(Is)] males and females (S2 Table).

All crosses between LR[*w*Pip(Is)] males and field females from the 19 localities were incompatible and displayed total embryonic mortality (N = 1452 egg rafts, Table 1). While confirming the strong sterilizing capacity of LR[*w*Pip(Is)] males towards field females sampled throughout La Réunion Island, this result also shows a temporal stability of CI intensity considering that the LR[*w*Pip(Is)] line has been maintained in the laboratory for over four years.

**Table 1:** Reciprocal crosses between the LR[*w*Pip(Is)] line and field mosquitoes from 19 localities in La Réunion.

Crosses	Phenotype of egg rafts (n)			Outcomes
	N	Infertile	Fertile	
♂ LR[*w*Pip(Is)] × ♀ St Philippe (#1)	35	100% (35)	0% (0)	BCI > UCI
♂ St Philippe (#1) × ♀ LR[*w*Pip(Is)]	20	80% (16)	20% (4)	
♂ LR[*w*Pip(Is)] × ♀ St Joseph (#2)	28	100% (28)	0% (0)	BCI > UCI
♂ St Joseph (#2) × ♀ LR[*w*Pip(Is)]	14	73% (11)	27% (3)	
♂ LR[*w*Pip(Is)] × ♀ St Pierre (#3)	73	100% (73)	0% (0)	BCI > UCI
♂ St Pierre (#3) × ♀ LR[*w*Pip(Is)]	92	78% (72)	22% (20)	
♂ LR[*w*Pip(Is)] × ♀ St Louis (#4)	77	100% (77)	0% (0)	BCI > UCI
♂ St Louis (#4) × ♀ LR[*w*Pip(Is)]	63	59% (37)	41% (26)	
♂ LR[*w*Pip(Is)] × ♀ Etang Salé (#5)	106	100% (106)	0% (0)	BCI > UCI
♂ Etang Salé (#5) × ♀ LR[*w*Pip(Is)]	58	79% (46)	21% (12)	
♂ LR[*w*Pip(Is)] × ♀ St Leu (#6)	53	100% (53)	0% (0)	BCI > UCI
♂ St Leu (#6) × ♀ LR[*w*Pip(Is)]	102	98% (100)	2% (2)	
♂ LR[*w*Pip(Is)] × ♀ St Gilles (#7)	102	100% (102)	0% (0)	BCI
♂ St Gilles (#7) × ♀ LR[*w*Pip(Is)]	77	100% (77)	0% (0)	
♂ LR[*w*Pip(Is)] × ♀ St Paul (#8)	84	100% (84)	0% (0)	BCI > UCI
♂ St Paul (#8) × ♀ LR[*w*Pip(Is)]	100	97% (97)	3% (3)	
♂ LR[*w*Pip(Is)] × ♀ Le Port (#9)	58	100% (58)	0% (0)	BCI > UCI
♂ Le Port (#9) × ♀ LR[*w*Pip(Is)]	77	68% (52)	32% (25)	
♂ LR[*w*Pip(Is)]× ♀ La Possession (#10)	74	100% (74)	0% (0)	BCI
♂ La Possession (#10) × ♀ LR[*w*Pip(Is)]	109	100% (109)	0% (0)	
♂ LR[*w*Pip(Is)] × ♀ St Denis (#11)	98	100% (98)	0% (0)	BCI > UCI
♂ St Denis (#11) × ♀ LR[*w*Pip(Is)]	97	87% (84)	13% (13)	
♂ LR[*w*Pip(Is)] × ♀ Ste Marie (#12)	78	100% (78)	0% (0)	BCI < UCI*
♂ Ste Marie (#12) × ♀ LR[*w*Pip(Is)]	95	26% (25)	74% (70)	
♂ LR[*w*Pip(Is)] × ♀ Ste Suzanne (#13)	82	100% (82)	0% (0)	BCI > UCI
♂ Ste Suzanne (#13) × ♀ LR[*w*Pip(Is)]	94	57% (54)	43% (40)	
♂ LR[*w*Pip(Is)] × ♀ St André (#14)	45	100% (45)	0% (0)	BCI > UCI
♂ St André (#14) × ♀ LR[*w*Pip(Is)]	42	93% (39)	7% (3)	
♂ LR[*w*Pip(Is)] × ♀ Bras Panon (#15)	113	100 (113)	0% (0)	BCI > UCI
♂ Bras Panon (#15) × ♀ LR[*w*Pip(Is)]	104	94% (98)	6% (6)	
♂ LR[*w*Pip(Is)] × ♀ St Benoît (#16)	87	100% (87)	0% (0)	BCI > UCI
♂ St Benoît (#16) × ♀ LR[*w*Pip(Is)]	134	90% (121)	10% (13)	
♂ LR[*w*Pip(Is)] × ♀ Ste Rose (#17)	93	100% (93)	0% (0)	BCI < UCI*
♂ Ste Rose (#17) × ♀ LR[*w*Pip(Is)]	118	19% (22)	81% (96)	
♂ LR[*w*Pip(Is)] × ♀ La Plaine des Palmistes (#18)	79	100% (79)	0% (0)	BCI > UCI
♂ La Plaine des Palmistes (#18) × ♀ LR[*w*Pip(Is)]	69	72% (50)	28% (19)	
♂ LR[*w*Pip(Is)] × ♀ La Plaine des Cafres (#19)	87	100% (87)	0% (0)	BCI < UCI*
♂ La Plaine des Cafres (#19) × ♀ LR[*w*Pip(Is)]	61	49% (30)	51% (31)	
**Total ♂ LR[*w*Pip(Is)] × ♀ La Réunion**	**1452**	**100% (1452)**	**0% (0)**	**BCI > UCI**
**Total ♂ La Réunion × ♀ LR[*w*Pip(Is)]**	**1526**	**75% (1140)**	**25% (386)**	

For each cross, the percentage of infertile egg rafts (all hatching rate (HR) = 0%) and fertile egg rafts (HR >90%) are reported. Outcomes correspond to the combination of reciprocal crosses between the LR[*w*Pip(Is)] line and field mosquitoes from each of the 19 localities. N = total number of egg rafts collected for each cross; BCI = bidirectionally incompatible crosses and UCI = unidirectionally incompatible crosses. *, localities where the percentage of UCI was higher than that of BCI. Localities are numbered as in Fig. 1.

In contrast to LR[*w*Pip(Is)] males, crosses between LR[*w*Pip(Is)] females and field males from the 19 localities produced polymorphic phenotypes with both infertile (HR = 0%) and fertile egg rafts (HR >90%) depending on crosses (Table 1). For instance when LR[*w*Pip(Is)] females were crossed with males from La Possession (#10), only infertile egg rafts were observed (N = 74); most egg rafts were infertile with males from St Denis (#11) [87% (N = 84/97)] whilst the frequency of infertile egg rafts was the lowest with males from Ste Rose (#17) [19% (N = 22/118)].

When considering crosses of the LR[*w*Pip(Is)] males and females with La Réunion field mosquitoes, two CI patterns were thus observed: bidirectional CI (or BCI *i*.*e*. only infertile egg rafts occurring in both reciprocal crosses between LR[*w*Pip(Is)] and field mosquitoes from a locality) and unidirectional CI (or UCI *i*.*e*. infertile egg rafts found in the cross between LR[*w*Pip(Is)] males and field females, and fertile egg rafts in the reciprocal cross). BCI was the most frequent CI pattern, occurring in all examined localities, with a frequency depending on localities (Table 1). Strict BCI was observed in two localities (in St Gilles, #7 and La Possession, #10). The frequency of BCI was higher than that of UCI in 14 localities (for instance in St Philippe, #1 and St Leu, #6) whilst the frequency of BCI was the lowest in three localities (Ste Marie, #12; Ste Rose, #17 and Plaine des Cafres, #19; Table 1).

### Performances of LR[wPip(Is)] males in field cages

**Fig 2:**
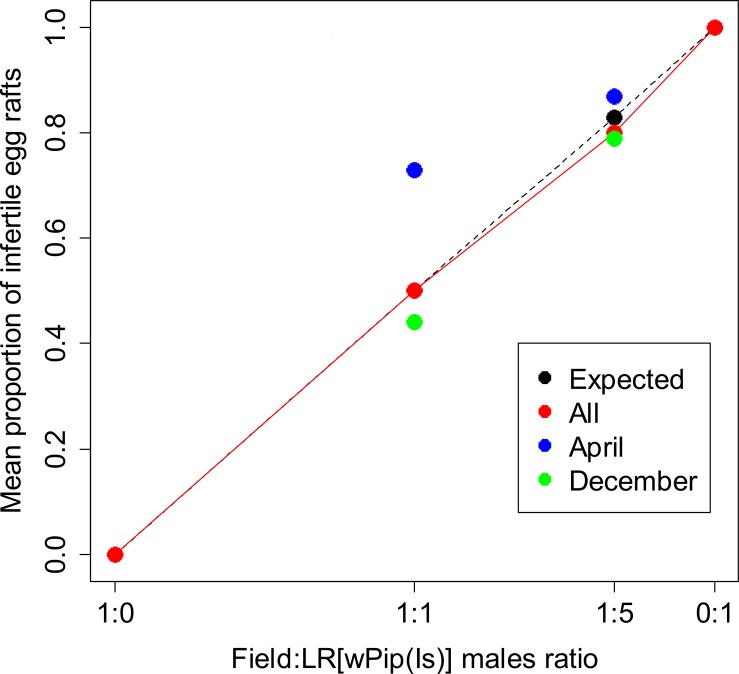
Assessment of mating competitiveness of LR[*w*Pip(Is)] males under semi-field conditions. In each confrontation, 200 field females were mixed with each of the four following field to LR[*w*Pip(Is)] males ratios: 1:0 (200 field males), 1:1 (200 field males and 200 LR[*w*Pip(Is)] males), 1:5 (200 field males and 1000 LR[*w*Pip(Is)] males) and 0:1 (200 LR[*w*Pip(Is)] males). The trials of the 1:0 and 0:1 ratios were performed in April; two trials for the 1:1 ratio were performed in April and four in December; whilst for the 1:5 ratio two trials were performed in April and three in December. Expected frequency of infertile egg rafts (in black) was calculated assuming equal competitiveness of LR[*w*Pip(Is)] and field males. Total embryonic mortality (HR = 0%) was noted in all infertile egg rafts.

As expected, all egg rafts from cages with only field males and females (1:0 ratio) were fertile (HR >90%) and full sterility (HR = 0%) occurred when only LR[*w*Pip(Is)] males were present with field females (0:1 ratio) (Fig. 2 and S1 Table). Experimental cages with distinct ratios of field to LR[*w*Pip(Is)] males (*i*.*e*. in 1:1 and 1:5 ratios) produced both fertile and infertile egg rafts. We never observed egg rafts with intermediate hatching rate, all egg rafts being either fully fertile or infertile.

Under random mating and equal males’ mating competitiveness hypotheses, equilibrated field to LR[*w*Pip(Is)] males ratio (1:1 ratio) should produce 50% infertile egg rafts whilst 83.3% (5/6) of infertile egg rafts are expected in the 1:5 field to LR[*w*Pip(Is)] males ratio. Observed frequencies of infertile egg rafts overall showed no significant deviation from theoretical expectations (Wilcox rank-sum's test: V = 49, *P* = 0.46); the mean frequency of infertile egg rafts produced by field females were 50% (N = 158 out of 313 egg rafts) and 80% (N = 148 out of 184 egg rafts) for the 1:1 and 1:5 ratios, respectively (Fig. 2 and S1 Table). The analysis of mating competitiveness according to experimental period (i.e. April and December) revealed higher frequencies of infertile egg rafts than expected in April compared to December where less infertile egg rafts were observed for both 1:1 and 1:5 ratios (Fig. 2). The frequencies of infertile egg rafts observed in the 1:1 ratio appeared higher than the expected frequency in all tests performed in April, this difference being significant in one of the two trials (Binomial exact test, *P* = 0.002 and *P* = 0.06 for the trials 1 and 2, respectively). In contrast to the results obtained in April, frequencies of infertile egg rafts observed in December were not significantly different from the expected frequency for three out of four trials. When the 1:5 ratio was implemented, no significant difference was noted between observed and expected frequencies of infertile egg rafts in April (Binomial exact test, all *P* > 0.3). However, in December, the observed frequencies of infertile egg rafts were either significantly higher (replication 3, Binomial exact test, *P* = 0.01) or significantly lower than expected (Binomial exact test, *P* = 0.01 and *P* = 0.03 for replications 4 and 5, respectively); however, significance did not resist the multiple Hommel’s sequential Bonferroni correction. A GLM model was then performed to test the effect of the experimental period (two-levels variable *i*.*e*. April and December) and ratio on the proportion of infertile egg rafts. Both variables had a significant effect (*P* < 0.0001).

**Fig 3:**
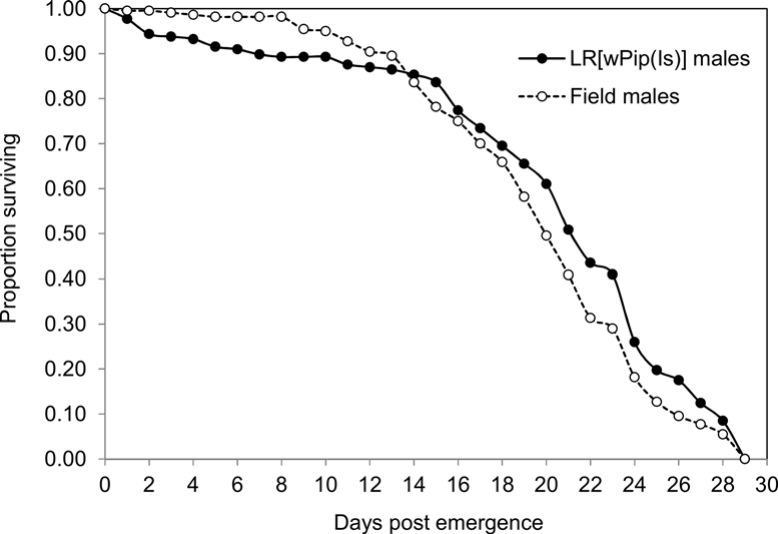
Survival curves of field males (N = 220; dotted line) and LR[*w*Pip(Is)] males (N = 177; solid line) in the semi-field setup.

Since males’ survival may affect mating competitiveness and thus sterilizing programs, we compared the survival of LR[*w*Pip(Is)] and field males. The overall survival of LR[*w*Pip(Is)] males recaptured from cages at the end of confrontations was not significantly different from that of field males, when considering all replicates or only the 1:1 ratio replicates (Kruskal-Wallis test, all: chi-squared = 0.176, df = 1, *P* = 0.67, 1:1 ratio: chi-squared = 0.10, df = 1, *P* = 0.75; Table 2). Moreover, no significant effect of the experimental period on males survival was noted (Kruskal-Wallis test, chi-squared = 3.57, df = 1, *P* = 0.06). Daily survival of field males (N = 220) and LR[*w*Pip(Is)] males (N = 177) was also investigated by counting dead males placed in separated cages in the semi-field setup. The survival of field males was higher than that of LR[*w*Pip(Is)] during the first 13 days of monitoring, whilst LR[*w*Pip(Is)] males survival was the highest from day 13 to day 28 (Fig. 3). However, when comparing the overall survival of both male categories, no significant difference was observed (χ^2^ = 2.78, *P* = 0.09, Fig. 3).

**Table 2:** Survival of LR[*w*Pip(Is)] and field males under semi-field conditions.

field♂:LR[*w*Pip(Is)]*♂* ratio	Period	Replicate		field♂			LR[*w*Pip(Is)]♂	
			Released	Recaptured	Mean recaptured ± SE	Released	Recaptured	Mean recaptured ± SE
1:0	April	1	200	26	12.5 ± 0.3	0	-	-
	April	2	200	24		0	-	-
0:1	April	1	0	-	-	200	107	57 ± 3.5
	April	2	0	-	-	200	121	
1:1	April	1	200	65	49.2 ± 10	200	146	46.7 ± 17.3
	April	2	200	12		200	4	
	December	3	200	70		200	79	
	December	4	200	150		200	115	
	December	5	200	136		200	100	
	December	6	200	158		200	116	
	Total		1600	641	40 ± 8.9	1600	788	49.2 ± 5

Each cage was set up by mixing 200 field females with different field to LR[*w*Pip(Is)] males ratios. Mosquitoes released in field cages were recaptured five days following releases. The survival was estimated by counting or by genotyping recaptured males after confrontations. All tests were performed in 2013.

## Discussion

The main purpose of this study was the examination of mating competitiveness of LR[*w*Pip(Is)] males under semi-field conditions. However, before performing tests in field cages, we first confirmed the sterilizing capacity of LR[*w*Pip(Is)] males through extensive crossing experiments with field females sampled in 19 localities in La Réunion Island. Indeed, due to the previously reported rapid evolution of CI phenotypes in the *Cx*. *pipiens* complex [31, 35, 42] extensive crossing experiments are required in order to: (i) assess sterilizing capacity of incompatible males towards an exhaustive number of field populations and (ii) control the stability of CI intensity over time.

Crosses between LR[*w*Pip(Is)] males and La Réunion field females showed that there was no alteration of sterilizing capacities of incompatible LR[*w*Pip(Is)] males in spite of four years of laboratory maintenance. However, crosses between LR[*w*Pip(Is)] females and field males revealed two phenotypes (compatibility and incompatibility) leading to both uni-and bidirectional CI phenotypes. This result complements previously reported data obtained with La Réunion mosquitoes sampled in a single locality St Denis (#11) in 2010 [4] and confirms that distinct crossing types do coexist within *Cx*. *quinquefasciatus* field populations in La Réunion Island [31]. These phenotypes may result from multiple *Wolbachia* infections, although they have never been evidenced in *Cx*. *pipiens* complex mosquitoes [27–29, 31]. In addition, there is no effect of *Wolbachia* density or nuclear background in the expression of CI in *Cx*. *pipiens* [31], in contrast to other host species such as *Drosophila* [43] or *Ae*. *albopictus* [44, 45]. The difference in crossing types of La Réunion mosquitoes have actually been previously proposed to be controlled by different *Wolbachia* infections, harbouring distinct *mod* and *resc* factors although these remain to be characterized [31, 35].

In addition to the sterilizing capacity of LR[*w*Pip(Is)] males, the success of an IIT vector control strategy will also depend on the ability of the released males to compete with indigenous wild males. So, intermediate tests under semi-field conditions are necessary to identify potential problems before proceeding to field implementation [46]. LR[*w*Pip(Is)] males exhibit mating competitiveness that is indistinguishable from field collected *Cx*. *quinquefasciatus* males, confirming the results observed under laboratory conditions showing comparable competitiveness of LR[*w*Pip(Is)] and La Réunion males [4]. An unexpected better competitiveness of LR[*w*Pip(Is)] males as compared to field males was observed in April (corresponding to the end of the warm humid season); whilst in December (*i*.*e*. at beginning of warm humid season), LR[*w*Pip(Is)] males displayed a mating competitiveness mostly comparable to that of wild males. Several factors including environmental conditions may affect mosquito quality and thus mating competitiveness of wild males in April. Larval rearing conditions such as density and food availability are known to modulate mosquito’s fitness [47–49]. The LR[*w*Pip(Is)] larvae were bred under low-crowding conditions and fed ad libitum, whilst larvae collected in natural breeding sites and used in the confrontations may have faced more challenging conditions. While LR[*w*Pip(Is)] line was reared under controlled conditions of the insectary, crowding and food availability faced by wild mosquitoes surely vary along the year but were not controlled in our experimental setup.

Finally, while CI and mating performances of incompatible males represent key parameters for implementing IIT, factors related to females such as mating choice of wild type females and accidental releases of females from the incompatible line can also affect the success of IIT. In this study, no evidence of mating choice was noted for field females, thus confirming random mating between *Cx*. *pipiens* mosquitoes infected by incompatible *Wolbachia* strains as previously reported [4, 24, 50–52]. For the accidental release of females, bidirectional CI between the incompatible line and specimens in target populations is expected to lower the risk of population replacement since incompatible females will be sterilized by field males. Data reported herein show that bidirectional CI is not the only CI phenotype observed in crosses between the LR[*w*Pip(Is)] line and La Réunion field mosquitoes; unidirectional CI also occurs and must be taken into consideration. Altogether, our data set encourages the development of an effective sexing method strictly producing LR[*w*Pip(Is)] males by any means (biological, genetic or transgenic, see [53–55]) in order to facilitate the operational up scaling of IIT together with minimizing the accidental release of LR[*w*Pip(Is)] females that would impair the success of such an attractive vector control strategy. Moreover, IIT as other sterile-male systems should be accompanied by regular molecular monitoring of field-caught mosquitoes in order to detect any impact of released mosquitoes on the evolution of natural populations. Although several studies have highlighted the potential of *Wolbachia* to inhibit pathogen replication [15–19]; *Wolbachia* can also increase pathogen replication as observed with both artificial [56] and natural [57] infections. So, vector competence analyses of LR[*w*Pip(Is)] females for pathogens of medical importance in the region such as lymphatic filarial and Rift Valley Fever virus are needed to fully investigate the impact of any accidental release of LR[*w*Pip(Is)] females in the field.

## Conclusion

Tests carried out in field cages in La Réunion Island confirm the stability of sterilizing performances of LR[*w*Pip(Is)] males and evidenced a good competitiveness of LR[*w*Pip(Is)] males compared to wild type males. In addition, crossing experiments confirm the coexistence of distinct CI phenotypes on several natural populations on the island, with limited but still not negligible expression of unidirectional CI. These data should be considered in future inundative releases as accidental releases of LR[*w*Pip(Is)] females without a proper monitoring of *Wolbachia* dynamics in natural populations might compromise the success of IIT.

## Supporting Information

S1 Fig Semi-field setup showing cages where mosquitoes were released (180×150×150 cm) covered with gardening tents (300×300×245 cm) used to prevent accidental escapes/invasions of mosquitoes and to protect the cages from rain. (PDF)

S2 Fig Mean temperatures (A) and relative humidity (B) in the selected site during the course of tests in field cages. (PDF)

S1 Table Competition cages with different ratios of LR[*w*Pip(Is)] males showing the number of infertile egg rafts amongst the total number of egg rafts collected in each trial. (DOC)

S2 Table Fertile crosses between mosquitoes from La Réunion collected in the localities Ste Marie (#12) and St André (#14); and between LR[*w*Pip(Is)] males and LR[*w*Pip(Is)] females. (DOC)
