# Supplementary material for: Wolbachia-Based Population Control Strategy Targeting Culex quinquefasciatus Mosquitoes Proves Efficient under Semi-Field Conditions
Source: PLoS One. 2015 Mar 13;10(3):e0119288. doi: 10.1371/journal.pone.0119288 (PMC4359102; doi:10.1371/journal.pone.0119288)
Supplement: S1 Fig — (PDF) [file pone.0119288.s001.pdf]

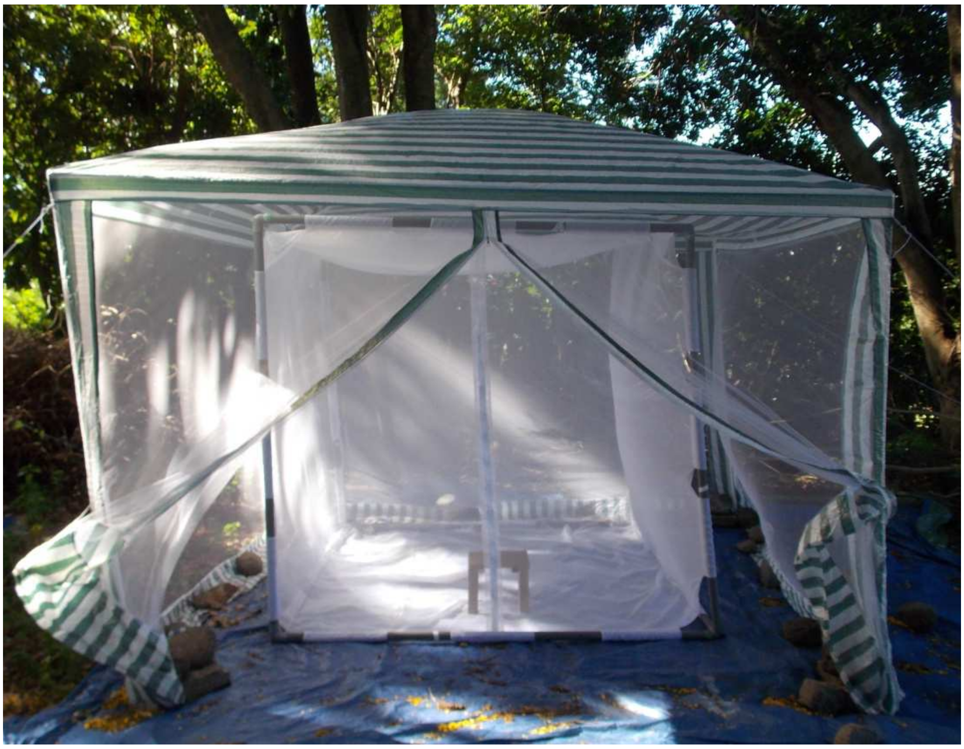

S1_Fig. Semi-field setup showing cages where mosquitoes were released (180×150×150 cm) covered with gardening tents (300×300×245 cm) used to prevent accidental escapes/invasions of mosquitoes and to protect the cages from rain.
