# Supplementary material for: Wolbachia-Based Population Control Strategy Targeting Culex quinquefasciatus Mosquitoes Proves Efficient under Semi-Field Conditions
Source: PLoS One. 2015 Mar 13;10(3):e0119288. doi: 10.1371/journal.pone.0119288 (PMC4359102; doi:10.1371/journal.pone.0119288)
Supplement: S2 Fig — (PDF) [file pone.0119288.s002.pdf]

A)

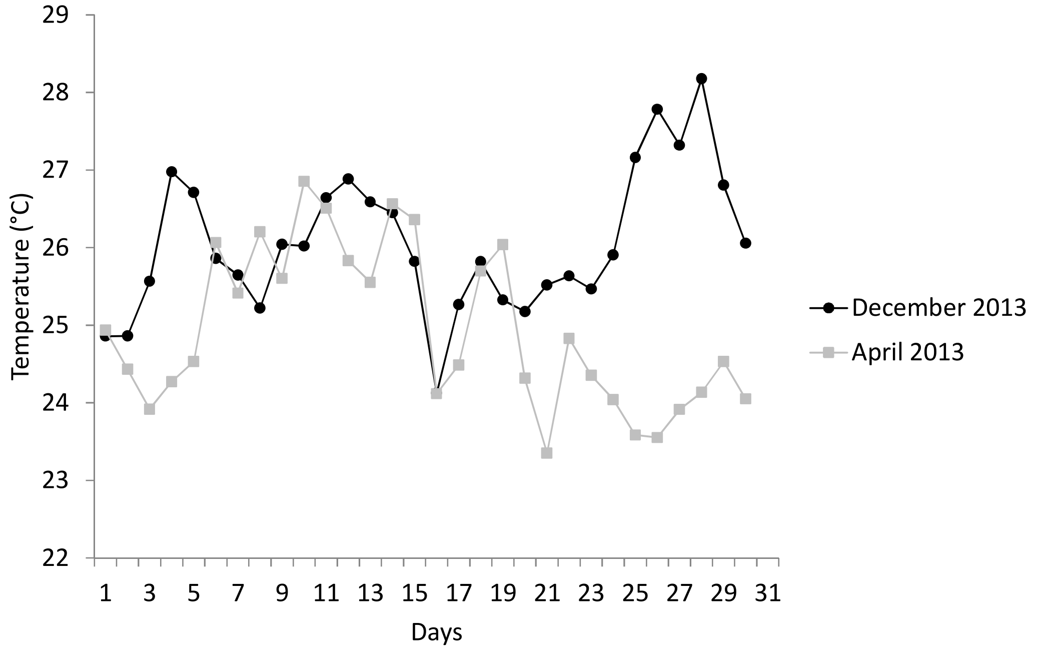

B)

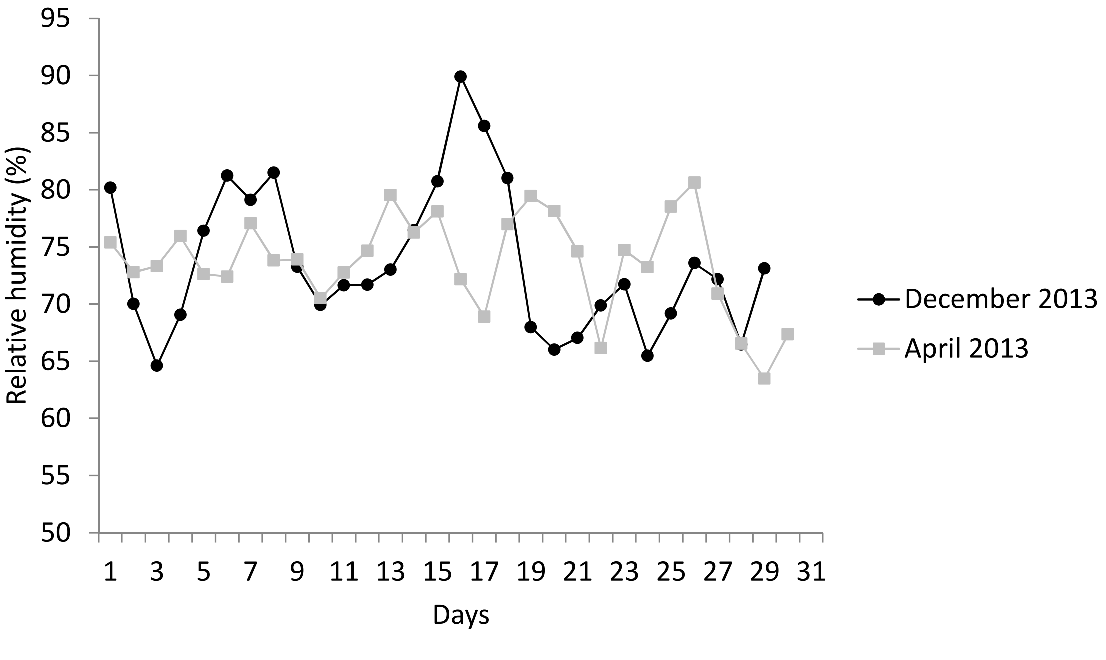

S2_Fig. Mean temperatures (A) and relative humidity (B) in the selected site during the course of tests in field cages.
