# Supplementary material for: Wolbachia-Based Population Control Strategy Targeting Culex quinquefasciatus Mosquitoes Proves Efficient under Semi-Field Conditions
Source: PLoS One. 2015 Mar 13;10(3):e0119288. doi: 10.1371/journal.pone.0119288 (PMC4359102; doi:10.1371/journal.pone.0119288)
Supplement: S1 Table — (DOC) [file pone.0119288.s003.doc]

**S1 Table.** Competition cages with different ratios of LR[*w*Pip(Is)] males showing the number of infertile egg rafts amongst the total number of egg rafts collected in each trial.

| Field♂:LR[*w*Pip(Is)]*♂* ratio |  | Number of males  ( field♂, LR[*w*Pip(Is)]♂) |  | Period |  | Trials |  | Number of egg rafts |  | Number of infertile egg rafts |
| --- | --- | --- | --- | --- | --- | --- | --- | --- | --- | --- |
| 1:0 |  | 200 (200, 0) |  | April 2013 |  | 1 |  | 25 |  | 0 |
|  |  |  |  | April 2013 |  | 2 |  | 10 |  | 0 |
| 0:1 |  | 200 (0, 200) |  | April 2013 |  | 1 |  | 11 |  | 11 |
|  |  |  |  | April 2013 |  | 2 |  | 20 |  | 20 |
| 1:1 |  | 400 (200, 200) |  | April 2013 |  | 1 |  | 48 |  | 35 |
|  |  |  |  | April 2013 |  | 2 |  | 19 |  | 14 |
|  |  |  |  | December 2013 |  | 3 |  | 60 |  | 30 |
|  |  |  |  | December 2013 |  | 4 |  | 90 |  | 33 |
|  |  |  |  | December 2013 |  | 5 |  | 61 |  | 29 |
|  |  |  |  | December 2013 |  | 6 |  | 35 |  | 17 |
| 1:5 |  | 1200 (200, 1000) |  | April 2013 |  | 1 |  | 18 |  | 17 |
|  |  |  |  | April 2013 |  | 2 |  | 12 |  | 9 |
|  |  |  |  | December 2013 |  | 3 |  | 49 |  | 47 |
|  |  |  |  | December 2013 |  | 4 |  | 42 |  | 28 |
|  |  |  |  | December 2013 |  | 5 |  | 63 |  | 47 |
