# Supplementary material for: Wolbachia-Based Population Control Strategy Targeting Culex quinquefasciatus Mosquitoes Proves Efficient under Semi-Field Conditions
Source: PLoS One. 2015 Mar 13;10(3):e0119288. doi: 10.1371/journal.pone.0119288 (PMC4359102; doi:10.1371/journal.pone.0119288)
Supplement: S2 Table — (DOC) [file pone.0119288.s004.doc]

**S2 Table.** Fertile crosses between mosquitoes from La Réunion collected in the localities Ste Marie (#12) and St André (#14); and between LR[*w*Pip(Is)] males and LR[*w*Pip(Is)] females.

|  | Males |  |  |
| --- | --- | --- | --- |
| Females | Ste Marie (#12) | St André (#14) | LR[*w*Pip(Is)] |
| Ste Marie (#12) | 23 | - | - |
| St André (#14) | - | 20 | - |
| LR[*w*Pip(Is)] | - | - | 15 |

For each cross, 50 males and 50 females were used. All mosquitoes were 2-5 days old and virgins. The numbers indicate the total number of egg rafts collected. All egg rafts collected showed a hatching rate > 90%. Dashes indicate that the crosses were not performed.
